# 28-homobrassinolide regulates antioxidant enzyme activities and gene expression in response to salt- and temperature-induced oxidative stress in *Brassica juncea*

**DOI:** 10.1038/s41598-018-27032-w

**Published:** 2018-06-07

**Authors:** Harpreet Kaur, Geetika Sirhindi, Renu Bhardwaj, M. N. Alyemeni, Kadambot H. M Siddique, Parvaiz Ahmad

**Affiliations:** 1 Department of Botany, Punjabi University, Patiala, 147002 Punjab India; 2 Department of Botanical & Environmental Sciences, GNDU, Amritsar, Punjab India; 3 Department of Botany and Microbiology, Faculty of Science, King Saud University, Riyadh, 11451 Saudi Arabia; 4 The UWA Institute of Agriculture and UWA School of Agriculture & Environment, The University of Western Australia, LB 5005, Perth, WA 6001 Australia; 5 Department of Botany, S.P. College, Srinagar, 190001 Jammu and Kashmir India

## Abstract

Brassinosteroids (BRs) are a group of naturally occurring plant steroid hormones that can induce plant tolerance to various plant stresses by regulating ROS production in cells, but the underlying mechanisms of this scavenging activity by BRs are not well understood. This study investigated the effects of 28-homobrassinolide (28-HBL) seed priming on *Brassica juncea* seedlings subjected to the combined stress of extreme temperatures (low, 4 °C or high, 44 °C) and salinity (180 mM), either alone or supplemented with 28-HBL treatments (0, 10^−6^, 10^−9^, 10^−12^ M). The combined temperature and salt stress treatments significantly reduced shoot and root lengths, but these improved when supplemented with 28-HBL although the response was dose-dependent. The combined stress alone significantly increased H_2_O_2_ content, but was inhibited when supplemented with 28-HBL. The activities of superoxide dismutase (SOD), catalase (CAT), ascorbate peroxidase (APOX), glutathione reductase (GR), dehydroascorbate reductase (DHAR) and monodehydroascorbate reductase (MDHAR) increased in response to 28-HBL. Overall, the 28-HBL seed priming treatment improved the plant’s potential to combat the toxic effects imposed by the combined temperature and salt stress by tightly regulating the accumulation of ROS, which was reflected in the improved redox state of antioxidants.

## Introduction

Temperature is a major environmental factor that affects plant growth and development. As sessile organisms, plants must be able to sense transient fluctuations as well as seasonal changes in temperature and respond to these changes by actively adjusting their biology to fit the subsequent temperature regime^1,2^. Farmers use various chemical fertilizers to improve plant growth and development, but their imbalanced use can hinder the growth of plants under such stresses^3^. Extensive chemical use increases the accumulation of different ions in the soil and leads to ionic stress, which is further enhanced by extreme climatic changes, particularly temperature fluctuations. Individual as well as combined stress conditions can cause imbalances in the homeostasis of the cell due to the overproduction of reactive oxygen species (ROS) such as superoxide radical (O^2−^), hydrogen peroxide (H_2_O_2_) and hydroxyl radical (OH^−^). ROS cause membrane deterioration, lipid peroxidation and DNA modifications that lead to irreparable metabolic and structural dysfunction and end in cell death^4^. To cope with ROS and maintain redox homeostasis, plants have a well-integrated antioxidant defense system—made up of antioxidant molecules and enzymes such as superoxide dismutase, catalase and those involved in the ascorbate–glutathione (AsA–GSH) cycle^4^-to manage any stress^5,6^. The high efficiency of these antioxidant enzymes can alleviate oxidative damage under abiotic stress^5–8^.

The AsA–GSH cycle is the central player in the antioxidant system in plant cells, operating mainly in the chloroplasts, which helps to prevent oxidative damage^9^ and maintain the photosynthetic efficiency of plants under stress. Ascorbate plays a role in the detoxification of ROS by reducing H_2_O_2_ to water^10^, a reaction that is catalyzed by ascorbate peroxidase (APOX, EC 1.11.1.11) and the reaction also generates monodehydroascorbate radicals, which are further reduced by NADPH in a reaction catalyzed by monodehydroascorbate reductase (MDHAR, EC 1.6.5.4)^11^. Monodehydroascorbate reductase radicals can spontaneously disproportionate to dehydroascorbate (DHA) and AsA^12^. The DHA reduction may occur via a non-enzymatic reaction with GSH or an enzymatic reaction with dehydroascorbate reductase (DHAR, EC 1.8.5.1) to produce AsA^13^. Recycling of GSH is catalyzed by glutathione reductase (GR, EC 1.6.4.2), which reduces glutathione disulfide (GSSG) by using NADPH^14^. Maintaining high ratios of GSH/GSSG and AsA/DHA are important for temperature and salt tolerance^14^. The coordinated regulation of these enzymatic reactions with external cues as well as endogenous chemicals is necessary for plant management under abiotic stress and is inextricably linked with phytohormones^15^.

Brassinosteroids (BRs) are polyhydroxylated plant steroid hormones that play a crucial role in cell division, gene expression, protein synthesis, photosynthesis and stress responses in plants^16,17^. 28-homobrassinolide (28-HBL) can induce plant tolerance to a variety of abiotic stresses including high and low temperatures, drought and salinity^5,18–24^. Serna, *et al*.^22^ analyzed the effects of BRs on alleviating salt stress in lettuce plants, where they were involved in the partial reversal of NaCl accumulation in plant cells. In *Brassica juncea* L., 28-HBL reduced the effects of salt stress by regulating photosynthetic efficiency and seed yield^25^. In *Lycopersicum esculentum*, BRs alleviated heat-induced inhibition of photosynthesis, increased carboxylation efficiency and enhanced the antioxidant system^26,27^. Brassinosteroids protect plants from germination to maturity by the up- and down-regulation of various non-enzymatic and enzymatic activities, including protein synthesis and accumulation, at the cellular level^28^ as well as the up-regulated expression of some components of translational machinery^29^. How 28-HBL interacts within plants during a combined stress of extreme temperature and salinity and how plants manage their physio-biochemical environment to combat the associated changes in cells, is unknown. The present study investigated the role of 28-HBL on growth and physio-biochemical processes in *B. juncea* by considering (i) the phenotypic performance of plants under combined stress of extreme temperature and salinity and the changes made by 28-HBL, (ii) the effect of the combined stress on cell viability and electrolyte leakage and their modifications by 28-HBL, (iii) 28-HBL inhibited H_2_O_2_ and MDA accumulation and (iv) the role of 28-HBL in altering antioxidant defense system components and the expression of stress tolerant genes. The study explored the impact of 28-HBL on growth, cell viability, electrolyte leakage, cellular activities of the antioxidant defense system and gene expression regulation in *B. juncea* L.to mitigate the toxic effects of combined stress of extreme temperature and salinity.

## Materials and Methods

### Plant material and treatments

Seeds of *B. juncea* L. cv. ‘RLC-1’ (certified) were obtained from the Department of Plant Breeding, Punjab Agriculture University, Ludhiana, India. Seeds were sterilized with 0.5% sodium hypochlorite for 15 minutes followed by 4–5 rinses in double-distilled water (DDW). The sterilized seeds were presoaked in various concentrations of 28-HBL (Control, 10^−6^,10^−9^, 10^−12^ M) for 8 h then germinated on autoclaved glass Petri dishes lined with Whatman No. 1 filter paper containing 0 or 180 mM NaCl salt for seven days. The 7-day-old seedlings were exposed to 4 °C or 44 °C temperature shocks for 5 h daily for three days. After the temperature shock treatments, the seedlings were transferred to a seed germinator in controlled laboratory conditions set at 24 °C, 16/8 h dark/light periods, uniform light fall on each Petri plate at 200 PAR and 70% humidity. The treatment details are as follows:

BR pre-soaking only: 0, 10^−6^, 10^−9^, 10^−12^ M (no salt or temp treatment)

Salt + BR: 180 mM + 0 M, 180 mM + 10^−6^ M, 180 mM + 10^−9^ M, 180 mM + 10^−12^ M (no temp treatment)

Low temp + BR pre-soak: 4 °C + 0 M, 4 °C + 10^−6^ M, 4 °C + 10^−9^ M, 4 °C + 10^−12^ M (no salt treatment)

High temp + BR pre-soak: 44 °C + 0 M, 44 °C + 10^−6^ M, 44 °C + 10^−9^ M, 44 °C + 10^−12^ M (no salt treatment)

Low temp + salt + BR pre-soak: 4 °C + 180 mM + 0 M, 4 °C + 180 mM + 10^−6^ M, 4 °C + 180 mM + 10^−9^ M, 4 °C + 180 mM + 10^−12^ M (salt and low temp treatment)

High temp + salt + BR pre-soak: 44 °C + 180 mM + 0 M, 44 °C + 180 mM + 10^−6^ M, 44 °C + 180 mM + 10^−9^ M, 44 °C + 180 mM + 10^−12^ M (salt and high temp treatment).

The low (4 °C) and high (44 °C) temperature treatments were selected based on the average temperature fluctuation from September to March in Punjab (India), being 2 °C ± 2 to 42 °C ± 2. We selected 4 °C and 44 °C after preliminary experiments on seed germination and initial seedling growth in *B. juncea* under controlled laboratory conditions.

To determine the sub-lethal concentration of NaCl, experiments were repeated five times under controlled conditions in a plant growth chamber; 180 mM NaCl was selected based on the IC_50_ (inhibition concentrations, where the growth of a plant remains 50%) for various morphological results of *B. juncea* L. cv. RLC-1 grown at 30, 60, 90, 120, 150, 180 and 200 mM NaCl.

Laboratory experiments were conducted five times under controlled conditions in a plant growth chamber to select the best concentration of 28-HBL for seedling growth and development in *B. juncea* L. cv. RLC-1. A concentrated stock solution of 28-HBL (10^−4^ M) was made, which was diluted to 10^−6^, 10^−9^ and 10^−12^ M for testing; 10^−9^ M 28-HBL was selected based on the results.

### Growth measurement

Twenty seedlings from each treatment were sampled randomly on the 10^th^ day for shoot and root lengths, measured with a ruler (cm).

### Content of hydrogen peroxide (H2O2) and Malondialdehyde (MDA), Electrolyte leakage, Cell viability assay and Cell non-viability assay estimation

The procedure of Velikova, *et al*.^30^ was used to estimate H_2_O_2_ content using a standard curve of known concentrations and expressed as µg g^−1^ FW.

MDA content was determined according to the methods described by Heath and Packer^31^. One milliliter of extract was added to 2 mL of a reaction solution containing 20% (v/v) trichloroacetic acid and 0.5% (v/v) thiobarbituric acid. The solution was placed in a water bath at 95 °C for 30 minutes before transferring to an ice water bath. The solution was centrifuged at 10,000 rpm for 10 minutes and the absorbance of the supernatant recorded at 532 and 600 nm.

The method of Lutts^32^ was used to estimate electrolyte leakage (EL). Electrical conductivity (L1) was recorded with a conductivity meter (PCS Testr35). The samples were then autoclaved at 120 °C for 20 minutes and electrical conductivity recorded (L2) after equilibration at 25 °C.

The method of Yang^33^ was used to determine cell viability in plant roots. Cell viability assays can be performed with fluorescein diacetate (FDA). Fluorescence was measured at 488–494 nm using a microscope (Nikon A1R Confocal Laser Scanning Microscope, Japan).

For the cell non-viability assay, the method of Truernit and Haseloff^34^ was used. Fluorescence was recorded at 535–617 nm using a confocal microscope (Nikon A1R Confocal Laser Scanning Microscope, Japan).

### Enzyme assays

One gram of leaf material was harvested and crushed in a pre-chilled pestle and mortar using 3 mL of 100 mM potassium phosphate buffer (PPB) (v/v) at pH 7.0. The crushed material was subjected to centrifugation using a cooling centrifuge for 20 minutes at 13,000 rpm at 4 °C. The supernatant was collected for total protein and various antioxidant enzyme analyses.

For total protein content, the method of Lowry, *et al*.^35^ was used. The optical density (OD) was recorded at 750 nm with a spectrophotometer (Shimadzu UV Mini 1240) using bovine serum albumin as a control.

*Superoxide dismutase (EC 1.15.1.1)* activity was estimated according to the method of Kono^36^, which is based on the principle of the inhibitory effect of SOD on the reduction of nitroblue tetrazolium (NBT) dye by superoxide radicals, which are generated by auto-oxidation of hydroxylamine hydrochloride. The reaction mixture, containing 1.3 mL sodium carbonate buffer (w/v), 500 μL NBT (w/v) and 100 μL Triton X-100 (v/v), was added to the test cuvettes. The reaction was initiated by adding 100 μl (w/v) hydroxylamine hydrochloride. After 2 minutes, 70 μL of the enzyme extract was added. The percent inhibition at the rate of NBT reduction was recorded as an increase in absorbance at 540 nm.

*Catalase (EC 1.11.1.6)* activity was measured according to Aebi^37^ by taking 3 mL of reaction mixture containing 100 mM phosphate buffer (v/v) at pH 7.0, 150 mM H_2_O_2_ (v/v) and 100 µL enzyme extract. The reaction was started by adding H_2_O_2_. CAT activity was measured as the decrease in absorbance at 240 nm for 30 s. Enzyme activity was computed using an extinction coefficient of 6.93 × 10^−3^ mM cm^−1^.

*Ascorbate peroxidase (EC 1.11.1.11)* activity was measured following the method of Nakano and Asada^38^ by monitoring the rate of decrease in absorbance at 290 nm for 1 minute. The reaction mixture contained 50 mM phosphate buffer (w/v) at pH 7.0, 5.0 mM ascorbate (w/v), 1.0 mM H_2_O_2_ (v/v) and 100 µL enzyme extract. Enzyme activity was calculated using an extinction coefficient of 2.8 mM cm^−1^.

*Glutathione reductase (EC 1.8.1.7)* activity was measured according to Carlberg and Mannervik^39^. The reaction mixture contained 1.5 mL of 50 mM phosphate buffer (w/v) at pH 7.0, 3 mM EDTA (w/v), 0.1 mM NADPH (w/v), 1 mM GSSG (w/v) and 600 µL enzyme extract. GR activity was calculated using an extinction coefficient of 6.22 mM cm^−1^ for NADPH at 340 nm for 1 minute.

*Monodehydroascorbate reductase (EC 1.6.5.4)* activity was assayed at 25 °C according to Hossain, *et al*.^40^. The reaction mixture contained 50 mM Tris-HCl buffer (pH 7.6), 0.125% Triton X-100 (v/v), 0.2 mM NADH (w/v), 2.5 mM ascorbate (w/v), 5 mg ascorbate oxidase and 100 µl enzyme extract. The decrease in absorbance at 340 nm due to NADH H^+^ oxidation (E = 6.2 mM cm^−1^) was monitored.

*Dehydroascorbate reductase (EC 1.8.5.1)* activity was assayed at 25 °C according to Dalton, *et al*.^41^. The 3 mL reaction mixture was prepared by mixing 50 mM potassium phosphate buffer (pH 7.0), 0.2 mM dehydroascorbate (w/v), 0.1 mM EDTA (w/v), 2.5 mM reduced glutathione (GSH) and 100 µl enzyme extract. DHAR activity was measured by following the increase in absorbance at 265 nm using an extinction coefficient of 14.0 mM cm^−1^.

The specific enzyme activity for all enzymes was expressed as unit mg^−1^ protein.

### RNA isolation and quantitative real-time PCR (qRTPCR)

Total RNA was isolated from the leaves of stressed and control samples with a RaFlex^TM^ solution as per the instructions (GeNei, India) and quantified spectrophotometrically. For real-time PCR, 5 µg of total RNA, 1 µl oligo (dT18) primer (0.5 µg µl^−1^) and 12 µl DEPC-treated water were used to make the first strand of cDNA using Revert AidTM RNAse H minus cDNA synthesis kit as per manufacturer’s instructions (Fermentas Life Sciences, USA). Other components included 200 u μl^−1^ Revert Aid TM M-MuLV Reverse Transcriptase, 20 u μl^−1^ RiboLockTM Ribonuclease Inhibitor, 100 µl 5 × Reaction Buffer, 10 mM dNTP, 0.5 µg µl^−1^ Oligo (dT)18 Primer and 0.5 µg µl^−1^ DEPC-treated water. Primers for real-time PCR were designed using MacVector 8.0 software (Table 1). The real-time PCR reaction was performed in a 20 µl reaction mixture containing diluted cDNA sample as a template and Power SYBR^®^ Green PCR master mix and 200 nM each of forward and reverse gene specific primers (Sigma-Aldrich St. Louis, MO). The reaction was performed using Step One^TM^ real-time PCR System (Applied Biosystems) with the following program: 95 °C (90 s) [94 °C (30 s), 55 °C (30 s), 72 °C (30 s)] × 40 cycles. To normalize the variance in RNA quality and cDNA input, the β-actin gene was used as an internal control in each case^42^. The C_t_ values of samples in different RNA samples were normalized with Ct values of β-actin. The relative expression ratio under stress conditions was calculated on an unstressed sample using REST 2005 version 1.9.12 software^43^.

**Table 1:** The sequence of forward and reverse primers size (20–25 bp) used in the gene expression analysis.

Sr. No.	Enzyme	Primer sequence	
		Forward 5′→3′	Reverse 3′→5′
1	**SOD**	5′GTCCACGCAGACCCTGATGT3'	3′GAAGACCAATAATACCGCAAGCA5'
2	**APOX**	5′CCGGTGAGAAGGAAGGTCTTC3′	3′CTTCCTCGTCAGCAGCGTATT5′
3	**CAT**	5′CTGACCCCCGCATCACA3′	3′ACGTTCAGACGGCTTGCAA5′
4	**DHAR**	5′CCAAAGGTGATGGGCTAAAGAG3′	3′ACATTGTACTAAAAGAAAGCAAGAGAAAAG5′
5	**MDHAR**	5′CCCAAAGCTAGCAAGAAGTCAAC3′	3′CTGTAGAGCGGCTTGAGCAA5′
6	**GR**	5′CACAGCAGCTGAGGAGTTTGTC3′	3′GACAGCTGTTTTAGCCTCAAGACTT5′
7	**Actin**	5′GGATCTCGAAGGGAGAGTACGA3′	3′TACCACACTCACCACCACGAA5′

### Statistical analysis

The statistical analysis was executed by one-way analysis of variance (ANOVA) to scrutinize interactions of NaCl (180 mM), 28-HBL (10^−6^, 10^−9^, 10^−12^ M) and temperature (4 °C, 44 °C) and expressed as mean ± SE (*n = *5). Tukey’s test (*P* ≤ 0.05) for the multiple comparisons using Graph Pad Prism version 5.04 was used to determine whether the means significantly differed from one another. The values represent the mean ± SE (*n = *5). *P* values ≤ 0.05 represent significant differences.

## Results

### 28-HBL inhibits dual stress of temperature and salinity by improving phenotypic expressions of B. juncea

**Figure 1:**
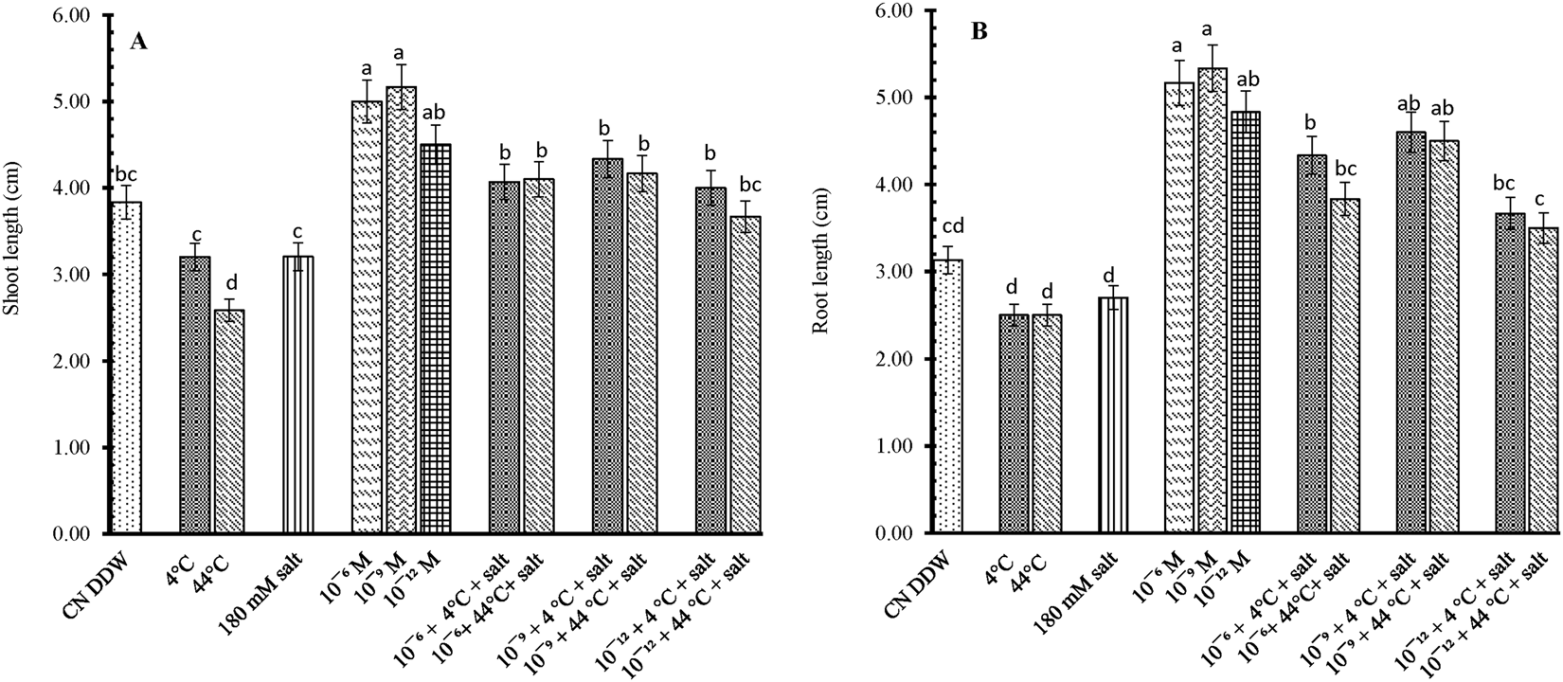
Effect of 28-HBL concentration (0, 10^−6^, 10^−9^, 10^−12^ M) on (**A**) shoot length and (**B**) root length of *B. juncea* L. under combinations of extreme temperature (4 °C, 44 °C) and NaCl salt (180 mM) stress in laboratory conditions. Different superscripted alphabetical letters within a column indicate significant differences from each other in all combinations (Tukey’s test, p ≤ 0.05).

The effect of 28-HBL on *B. juncea* L. growth is concentration dependent. Individual treatments of extreme temperatures of both low and high (4 °C, 44 °C) and salt (180 mM) decreased shoot lengths by 17.00, 33.00 and 17.00%, respectively, over the control seedlings of the same age. The 28-HBL primed seedlings tolerated extreme temperatures and salinity, when given either individually or in combination, better than untreated seedlings raised in DDW alone, as indicated by their enhanced cumulative shoot lengths (5.00 ± 0.01, 5.16 ± 0.16, 4.50 ± 0.02 vs. 3.83 ± 0.44 cm). Exogenous priming of 28-HBL treatments mitigate the negative effect of dual stress toxicity and 10^−9^ M treatment produced the longest shoots (5.16 ± 0.16 cm), with 34% record increase over untreated CN DDW (control treated with DDW only) seedlings (Fig. 1A).

The individual high- and low- temperature treatments reduced root length (RL) by 21% compared to CN DDW seedlings. The 180 mM NaCl further added 13.73% to the reduction in RL than the extreme temperature treatments, with the greatest reduction of 20.12% in CN DDW seedlings (Fig. 1B). The individual 28-HBL treatments enhanced RL the most in CN DDW seedlings. In all concentrations, root length increased by 5.16 ± 0.92 cm, 5.33 ± 0.44 cm and 4.83 ± 0.92 cm with 10^−6^ M, 10^−9^ M and 10^−12^ M 28-HBL, respectively.

Of the 28-HBL treatments, 10^−9^ M in control plants (without stress) produced the longest roots (5.33 ± 0.44 cm) which were 70% longer than the CN seedlings. This increase was much higher than the individual temperature- or salt-stressed seedlings. The ameliorative effect of 28-HBL in mitigating the stress effect on RL was observed in the combined treatments of 28-HBL and stress (temperature and/or salt). The best combination was 10^−9^ M 28-HBL + 4 °C + 180 mM NaCl where RL increased by 46% compared with the individual 4 °C treatment.

### Effect of 28-HBL, temperature and salt on hydrogen peroxide (H_2_O_2_), lipid peroxidation (MDA) and electrolyte leakage (EL)

**Figure 2:**
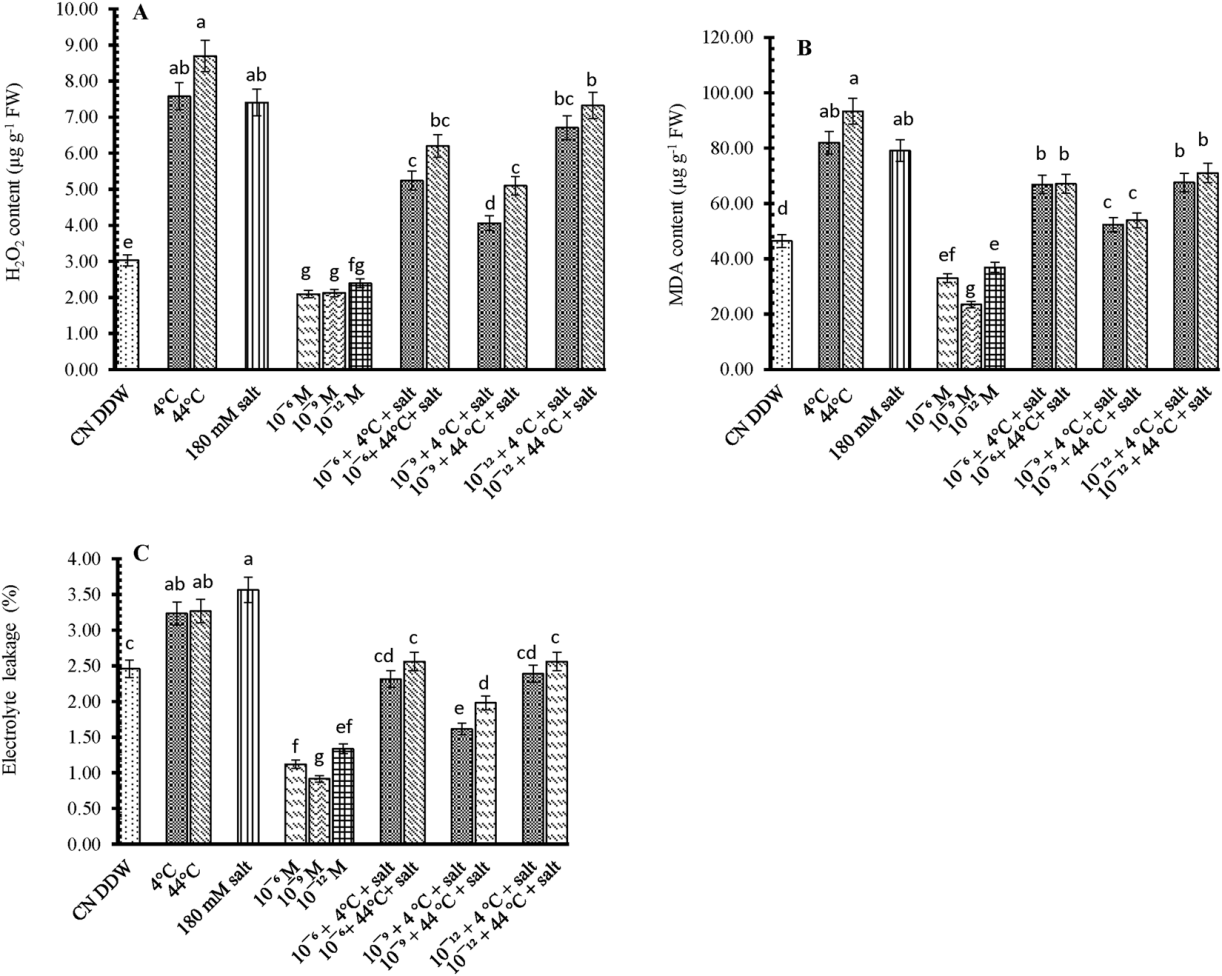
Effect of 28-HBL concentration (0, 10^−6^, 10^−9^, 10^−12^ M) on (**A**) hydrogen peroxide (H_2_O_2_) content, (**B**) MDA (lipid peroxidation) content and (**C**) electrolyte leakage (EL%) of *B. juncea* L. under combinations of extreme temperature (4 °C, 44 °C) and NaCl salt (180 mM) stress in laboratory conditions. Different superscripted alphabetical letters within a column indicate significant differences from each other in all combinations (Tukey’s test, p ≤ 0.05).

The hydrogen peroxide (H_2_O_2_) content increased in 10-day-old *B. juncea* seedlings exposed to individual treatments of extreme temperature (4 °C or 44 °C) or salt (180 mM NaCl) (Fig. 2A). Supplementation with 28-HBL (10^−6^, 10^−9^ and 10^−12^) reduced H_2_O_2_ levels by 31, 31 and 22%, respectively, compared with CN DDW seedlings. In seedlings exposed to combined temperature and salt stress, 28-HBL supplementation reduced H_2_O_2_ contents further, by 46.43% and 45.13% with 10^−9^ M 28-HBL + 4 °C + salt compared to individual treatments with 4 °C and salt respectively.

Individual treatments of extreme temperature (4 °C or 44 °C) or salt (180 mM NaCl) increased MDA content (Fig. 2B), more so at 44 °C (93.31 ± 0.55), being double that of control plants (46.45 ± 0.47). Exogenous application of 28-HBL regulated MDA content, more so at 10^−9^ M (23.44 ± 0.76), being half that of the control plants. Of the combined stress treatments, the 10^−9^ M + 4 °C + 180 mM salt treatment produced the lowest MDA content (52.32 ± 0.59), being 64 and 58% less than the 10^−9^ M + 4 °C (81.97 ± 0.96) and 10^−9^ M + 180 mM NaCl (79.11 ± 0.43) treatments, respectively and 26 and 20% less than the individual 4 °C (81.97 ± 0.96) and 180 mM NaCl (79.11 ± 0.43) treatments, respectively.

The rate of electrolyte leakage increased in 10-day-old seedlings exposed to 4 °C (3.23 ± 0.31), 44 °C (3.27 ± 0.29) and 180 mM NaCl (3.56 ± 0.12) compared to the untreated seedlings (2.46 ± 0.10) (Fig. 2C). Supplementation with 28-HBL reduced the rate of EL and, in 10^−9^ M treatment at normal (25 °C) temperature, EL was 37% less (0.92 ± 0.23) than the control seedlings. Electrolyte leakage was more in stressed seedlings (4 °C, 44 °C and salt) than CN DDW seedlings while those presoaked in 28-HBL had little electrolyte leakage. The temperature and salt-treated seedlings presoaked with 28-HBL had less electrolyte leakage than individual temperature or salt treatments but not to the same extent as the individual 28-HBL treatments. The 10^−9^ M 28-HBL + 4 °C + 180 mM NaCl treatment had the largest reduction in EL (35%), relative to the CN DDW seedlings, which was 4% better than the individual 4 °C treatment and 9% better than the individual 180 mM NaCl treatment.

### Effect of 28-HBL, temperature and salt on cell viability and non-viability assay

**Figure 3:**
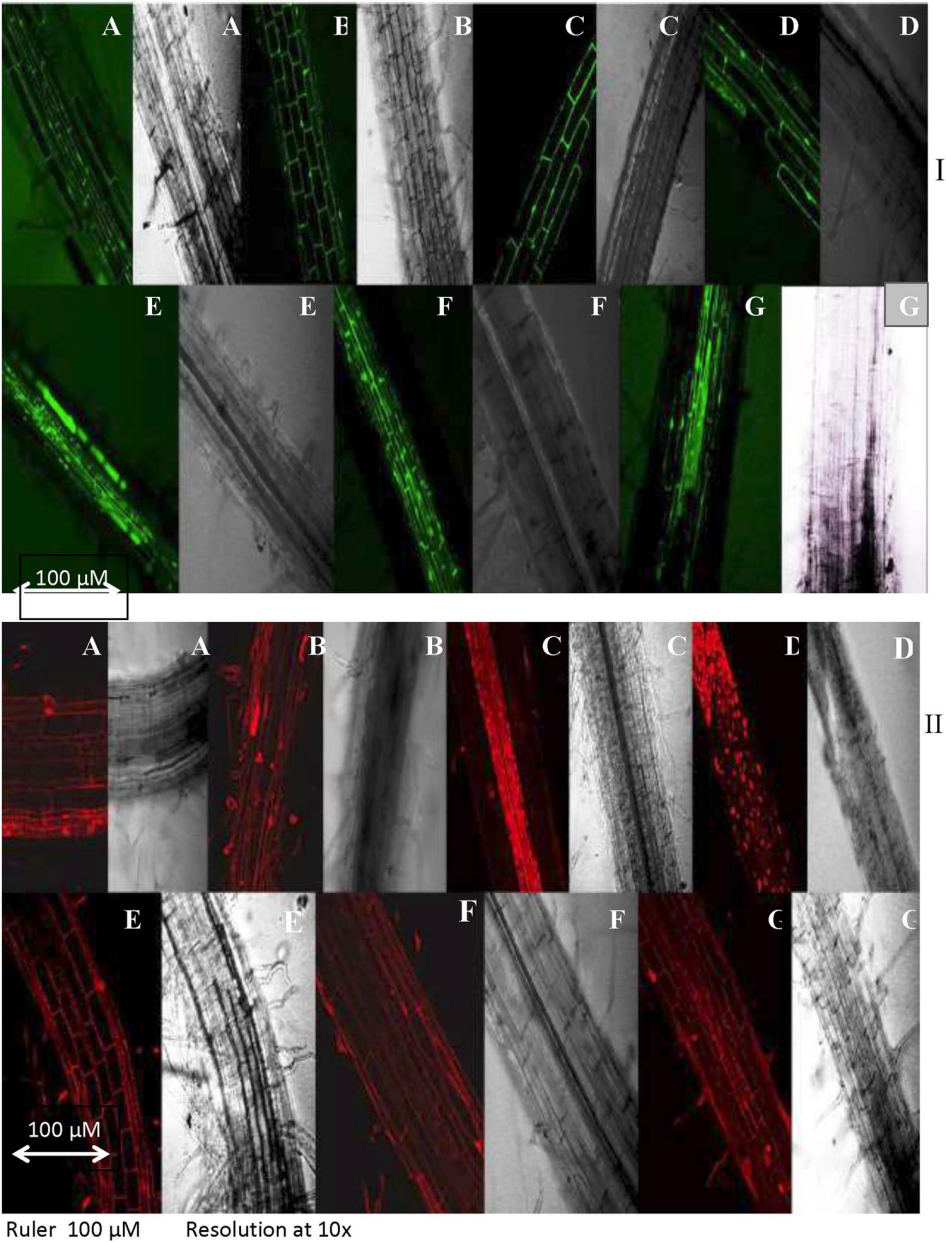
Effect of 28-HBL concentration (0, 10^−9^ M) on plant (I) cell viability assay and (II) cell non-viability assay of *B. juncea* L. under combinations of extreme temperature (4 °C, 44 °C) and NaCl salt (180 mM) stress in laboratory conditions. (**A**) control, (**B**) 4 °C, (**C**) 44 °C, (**D**) 180 mM NaCl, (**E**) 10^−9^ M 28-HBL, (**F**) 10^−9^ M + 4 °C + 180 mM NaCl and (**G**) 10^−9^ M + 44 °C + 180 mM NaCl.

Intracellular viability in temperature- and salt-stressed *B. juncea* L. seedlings (Fig. 3A) was detected using fluorescein diacetate (FDA) which fluoresces green when it reacts with viable cells. The cell membrane is permeable to FDA so it can enter cells freely. Area of viable cells in *B. juncea* seedlings raised in temperature (4 °C or 44 °C) and/or salt (180 mM NaCl) alone or in dual stress treatment, enhanced oxidative damage resulted in reducing viability which was shown as decrease in green color intensity under a confocal microscope. The increase in green fluorescence in 10^−9^ M 28-HBL treated seedlings with or without temperature and salt stress suggests that 28-HBL has the potential to modulate oxidative stress in *B. juncea* L.

The different temperature and/or salt stress treatments enhanced oxidative stress many-fold, which resulted in cell membrane disintegration that rendered cells non-viable or caused cell death. In the confocal studies, extreme temperature (4 °C or 44 °C) and/or salt supplemented with 28-HBL reduced cell non-viability to varying extents, as indicated by the intensity of the red color in damaged cells (Fig. 3B). Supplementation with 10^−9^ M 28-HBL regulated cell damage more efficiently and (reduced color intensity) than those seedlings treated with alone stress treatments and presoaked only with DDW control.

### Effect of 28-HBL, temperature and salt on total soluble protein content and antioxidant enzyme activities

**Figure 4:**
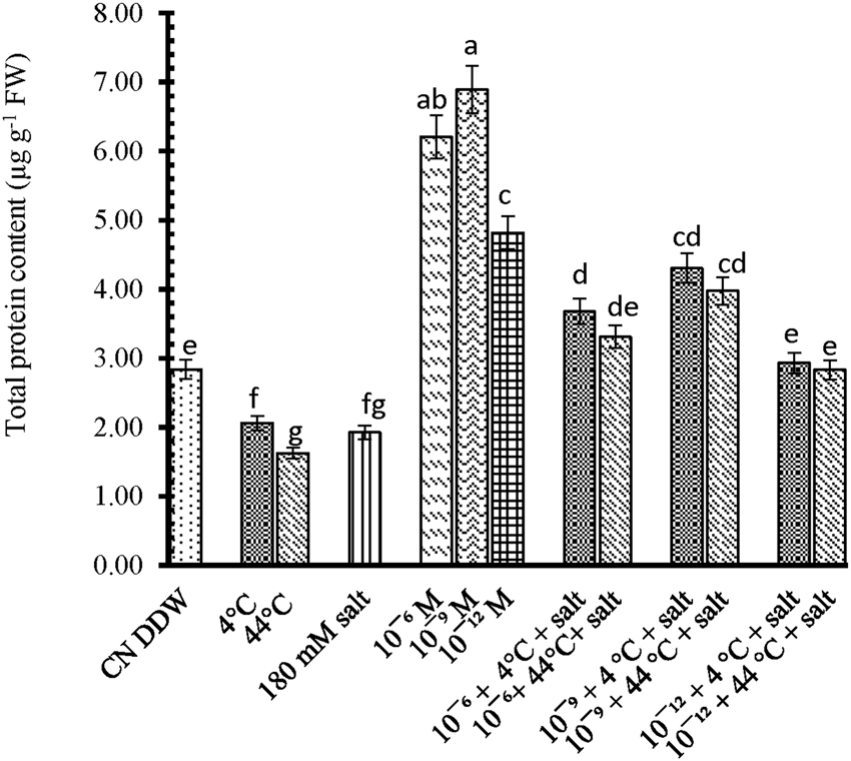
Effect of 28-HBL concentration (0, 10^−6^, 10^−9^, 10^−12^ M) on total soluble protein content of *B. juncea* L. under combinations of extreme temperature (4 °C, 44 °C) and NaCl salt (180 mM) stress in laboratory conditions. Different superscripted alphabetical letters within a column indicate significant differences from each other in all combinations (Tukey’s test, p ≤ 0.05).

Seedlings exposed to 4 °C, 44 °C and salt had 28, 43 and 33% less total soluble protein contents, respectively, than the control plants. The addition of 28-HBL to seedlings exposed to extreme temperature or salt stress reduced protein content less than those exposed to individual temperature or salt treatments (Fig. 4). Seedlings supplemented with 10^−9^ M 28-HBL enhanced soluble protein by 51% (4 °C + salt) and 40% (44 °C + salt) compared to the control.

SOD activity in seedlings of *B. juncea* L. increased by 34, 72 and 44% after exposure to 4 °C, 44 °C and 180 mM NaCl salt, respectively, compared with the control seedlings (Table 2). The individual 28-HBL treatments did not significantly increase SOD activity compared to control seedlings. However, SOD activity increased in seedlings exposed to combined temperature and salt stress and supplemented with 28-HBL, more so with 10^−9^ M 28-HBL. The highest SOD activity occurred in the 10^−9^ M 28-HBL + 44 °C + salt treatment, being 216% higher than 44 °C alone and 244% higher than the salt only treatment.

**Table 2:** Effect of 28-HBL concentrations (0, 10^−6^, 10^−9^, 10^−12^ M) on SOD, CAT and APOX activities of *B. juncea* L.

Treatments	SOD activity (U min^−1^ mg^−1^ proteins)	CAT activity (U min^−1^ mg^−1^ proteins)	APOX activity (U min^−1^ mg^−1^ proteins)
Control 4 °C 44 °C 180 mM NaCl 10^−6^ M 28-HBL 10^−9^ M 28-HBL 10^−12^ M 28-HBL 10^−6^ M + 180 mM + 4 °C 10^−6^ M + 180 mM + 44 °C 10^−9^ M + 180 mM + 4 °C 10^−9^ M + 180 mM + 44 °C 10^−12^ M + 180 mM + 4 °C 10^−12^ M + 180 mM + 44 °C	24.67 ± 0.32^j^ 32.98 ± 0.79^h^ 39.83 ± 0.98^f^ 36.36 ± 0.81^g^ 30.56 ± 0.27^i^ 32.68 ± 0.23^h^ 30.37 ± 0.27^i^ 71.32 ± 0.78^c^ 80.05 ± 1.00^b^ 80.90 ± 0.94^b^ 94.28 ± 1.24^a^ 46.64 ± 0.90^e^ 52.84 ± 1.16^d^	26.43 ± 0.46^g^ 38.00 ± 0.61^d^ 43.56 ± 0.76^c^ 39.03 ± 0.64^d^ 36.76 ± 0.61^de^ 36.66 ± 0.59^de^ 29.66 ± 0.50^f^ 47.13 ± 0.83^bc^ 50.73 ± 0.95^b^ 50.96 ± 1.04^b^ 66.66 ± 1.53^a^ 45.73 ± 0.64^c^ 45.96 ± 0.67^c^	18.82 ± 0.40^f^ 28.49 ± 0.28^d^ 30.42 ± 0.32^cd^ 29.52 ± 0.30^d^ 26.49 ± 0.23^e^ 27.71 ± 0.25^de^ 27.14 ± 0.24^e^ 33.31 ± 0.36^c^ 34.03 ± 0.42^c^ 36.13 ± 0.59^b^ 45.62 ± 0.84^a^ 31.51 ± 0.38^d^ 33.49 ± 0.40^c^
F-ratio_12×26_	1100	188.67	80.45

Under combinations of extreme temperature (4 °C, 44 °C) and NaCl salt (180 mM) stress in laboratory conditions.

The data represented above are means ± S.E (n = 5). Different superscripted alphabetical letters within a column indicate significant differences from each other in all combinations (Tukey’s test, p ≤ 0.05).

Catalase (CAT) activity in seedlings of *B. juncea* L. increased by 43, 64 and 47% after exposure to 4 °C, 44 °C and salt, respectively, compared with control seedlings (Table 2). The individual 28-HBL treatments triggered CAT activity-but to a lesser extent than seedlings exposed to individual temperature or salt stress-increasing by 39, 38 and 12%, respectively, compared with the control seedlings. Seedlings exposed to combined temperature and salt stress treatments had the highest CAT activity. The best combination was 10^−9^ M 28-HBL + 44 °C + salt with a 152% increase in CAT activity, relative to the controls.

APOX activity in seedlings of *B. juncea* L. increased by 51, 61 and 56% after exposure to individual 4 °C, 44 °C and salt treatments, respectively, compared with the control seedlings (Table 2). The individual 28-HBL treatments increased APOX activity almost to the same level as the control seedlings. Seedlings exposed to combined temperature and salt stress with 28-HBL supplementation recorded increased APOX activity. The best combination was 10^−9^ M 28-HBL + 44 °C + salt with APOX activity being 81% higher than the individual 44 °C treatment and salt only treatment.

GR activity in seedlings of *B. juncea* L. increased by 87, 102 and 91% after exposure to individual 4 °C, 44 °C and salt treatments, respectively, compared with the control seedlings (Table 3). The individual 28-HBL treatments enhanced GR activity, but not to the same extent as the individual temperature or salt stresses. The best individual 28-HBL treatment was 10^−9^ M with a 52% increase in GR activity compared with the control. Seedlings exposed to combined temperature and salt stress with 28-HBL recorded the highest GR activities. The best combination was 10^−9^ M 28-HBL + 44 °C + salt where GR activity increased by 254%, compared with control seedlings, 152% relative to 44 °C alone and 163% relative to the salt only treatment.

**Table 3:** Effect of 28-HBL concentrations (0, 10^−6^, 10^−9^, 10^−12^ M) on MDHAR, DHAR and GR activities of *B. juncea* L.

Treatments	GR activity (U min^−1^ mg^−1^ proteins)	MDHAR activity (U min^−1^ mg^−1^ proteins)	DHAR activity (U min^−1^ mg^−1^ proteins)
Control 4 °C 44 °C 180 mM NaCl 10^−6^ M 28-HBL 10^−9^ M 28-HBL 10^−12^ M 28-HBL 10^−6^ M + 180 mM + 4 °C 10^−6^ M + 180 mM + 44 °C 10^−9^ M + 180 mM + 4 °C 10^−9^ M + 180 mM + 44 °C 10^−12^ M + 180 mM + 4 °C 10^−12^ M + 180 mM + 44 °C	24.23 ± 0.14^l^ 45.21 ± 0.46^h^ 48.98 ± 0.55^g^ 46.20 ± 0.49^h^ 29.56 ± 0.21^j^ 36.91 ± 0.24^i^ 28.40 ± 0.18^k^ 64.97 ± 0.75^d^ 68.66 ± 0.84^c^ 72.31 ± 0.95^b^ 85.43 ± 1.11^a^ 53.00 ± 0.61^f^ 62.32 ± 0.71^de^	23.04 ± 0.32^e^ 29.12 ± 0.60^c^ 33.31 ± 0.71^b^ 30.35 ± 0.65^c^ 26.58 ± 0.54^d^ 27.17 ± 0.56^cd^ 24.09 ± 0.36^e^ 33.49 ± 0.72^b^ 34.36 ± 0.75^b^ 36.02 ± 0.80^ab^ 38.50 ± 0.88^a^ 28.74 ± 0.59^cd^ 30.60 ± 0.67^bc^	9.34 ± 0.10^j^ 14.37 ± 0.23^f^ 17.31 ± 0.29^d^ 15.71 ± 0.25^e^ 12.08 ± 0.15^h^ 13.59 ± 0.18^fg^ 11.36 ± 0.14^i^ 19.13 ± 0.34^c^ 20.61 ± 0.36^b^ 21.90 ± 1.39^ab^ 24.10 ± 0.45^a^ 18.31 ± 0.30^cd^ 18.76 ± 0.32^c^
F-ratio_12×26_	417.4	60.58	49.20

Under combinations of extreme temperature (4 °C, 44 °C) and NaCl salt (180 mM) stress in laboratory conditions.

The data represented above are means ± S.E (n = 5). Different superscripted alphabetical letters within a column indicate significant differences from each other in all combinations (Tukey’s test, p ≤ 0.05).

Monodehydroascorbate reductase (MDHAR) activity in seedlings of *B. juncea* L. increased by 26, 44 and 31% after exposure to individual 4 °C, 44 °C and salt treatments, respectively, compared with the control seedlings (Table 3). The individual 28-HBL treatments enhanced MDHAR activity, but not to the same extent as the individual temperature or salt stresses. The best individual 28-HBL treatment was 10^−9^ M with a 17% increase in MDHAR activity compared with the control. Seedlings exposed to combined temperature and salt stress with 28-HBL recorded the highest MDHAR activities. The best combination was 10^−9^ M 28-HBL + 44 °C + salt, where MDHAR activity increased by 67% relative to the control.

DHAR activity in seedlings of *B. juncea* L. increased by 53, 85 and 68% after exposure to individual 4 °C, 44 °C and salt treatments, respectively, compared with the control seedlings (Table 3). The individual 28-HBL treatments enhanced DHAR activity, but not to the same extent as the individual temperature or salt stresses. The best individual 28-HBL treatment was 10^−9^ M with a 45% increase in DHAR activity compared with the control. Seedlings exposed to combined temperature and salt stress with 28-HBL recorded the highest DHAR activities. The best combination was 10^−9^ M 28-HBL + 44 °C + salt, where DHAR activity increased by 157% relative to the 10^−9^ M 28-HBL only treatment.

### Effect of 28-HBL, temperature and salt on antioxidant gene expression

**Figure 5:**
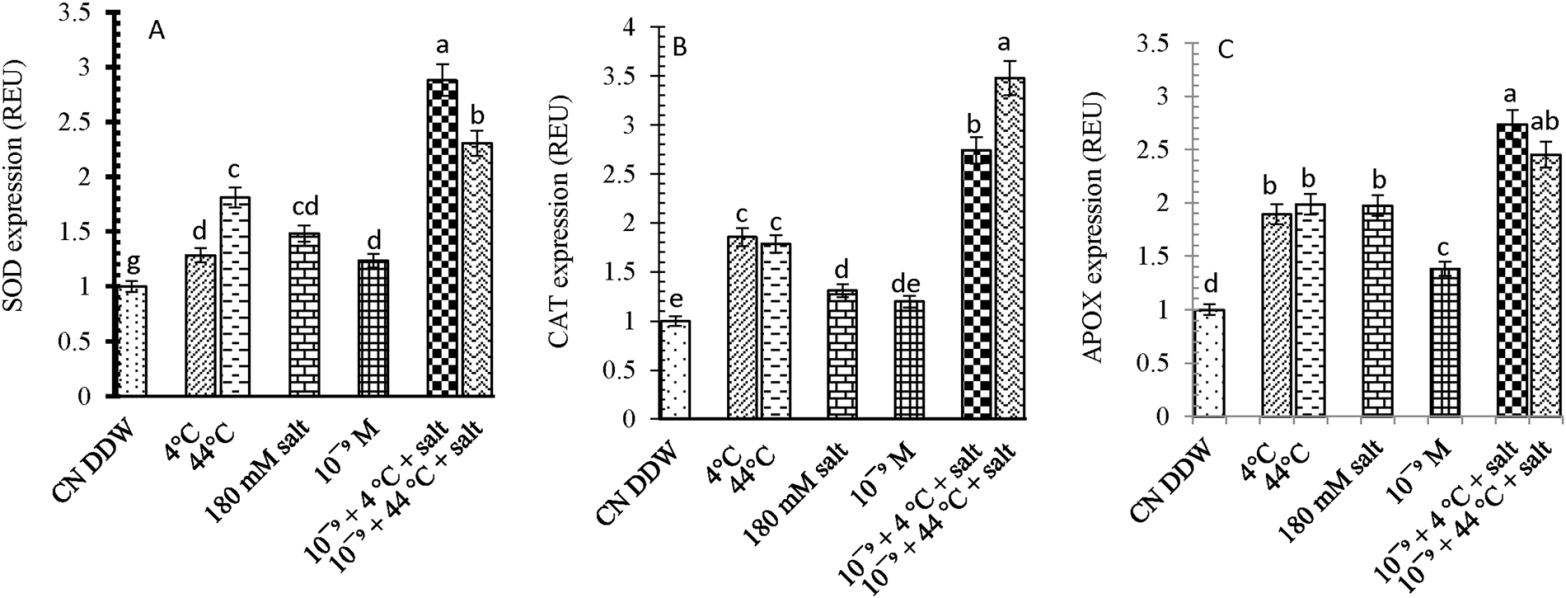
Effect of 28-HBL concentration (0, 10^−9^ M) on (**A**) SOD, (**B**) CAT and (**C**) APOX genes (REU relative expression unit) of *B. juncea* L. under combinations of extreme temperature (4 °C, 44 °C) and NaCl salt (180 mM) stress in laboratory conditions. Different superscripted alphabetical letters within a column indicate significant differences from each other in all combinations (Tukey’s test, p ≤ 0.05).

SOD expression in seedlings of *B. juncea* L. increased by 28, 80 and 48% after exposure to individual 4 °C, 44 °C and salt treatments, respectively, compared with the control seedlings (Fig. 5A). The pre-treatment with 28-HBL further enhanced SOD expression by 188% with 10^−9^ M 28-HBL + 4 °C + salt and 130% with 10^−9^ M 28-HBL + 44 °C + salt, compared to the control.

CAT expression in seedlings of *B. juncea* L. was up-regulated by 85, 78 and 30% after exposure to individual 4 °C, 44 °C and salt treatments, respectively, compared with the control seedlings (Fig. 5B). The individual 10^−9^ M 28-HBL treatment increased CAT expression by 19%, which was further up-regulated by 174% and 247% with 4 °C + salt and 44 °C + salt, respectively, relative to the control.

APOX expression in seedlings of *B. juncea* L. was up-regulated by 89, 98 and 97% after exposure to individual 4 °C, 44 °C and salt treatments, respectively, compared with the control seedlings (Fig. 5C). The individual 10^−9^ M 28-HBL treatment increased APOX expression by 47%, which was further up-regulated by 173% and 142% with 4 °C + salt and 44 °C + salt, respectively, relative to the control.

**Figure 6:**
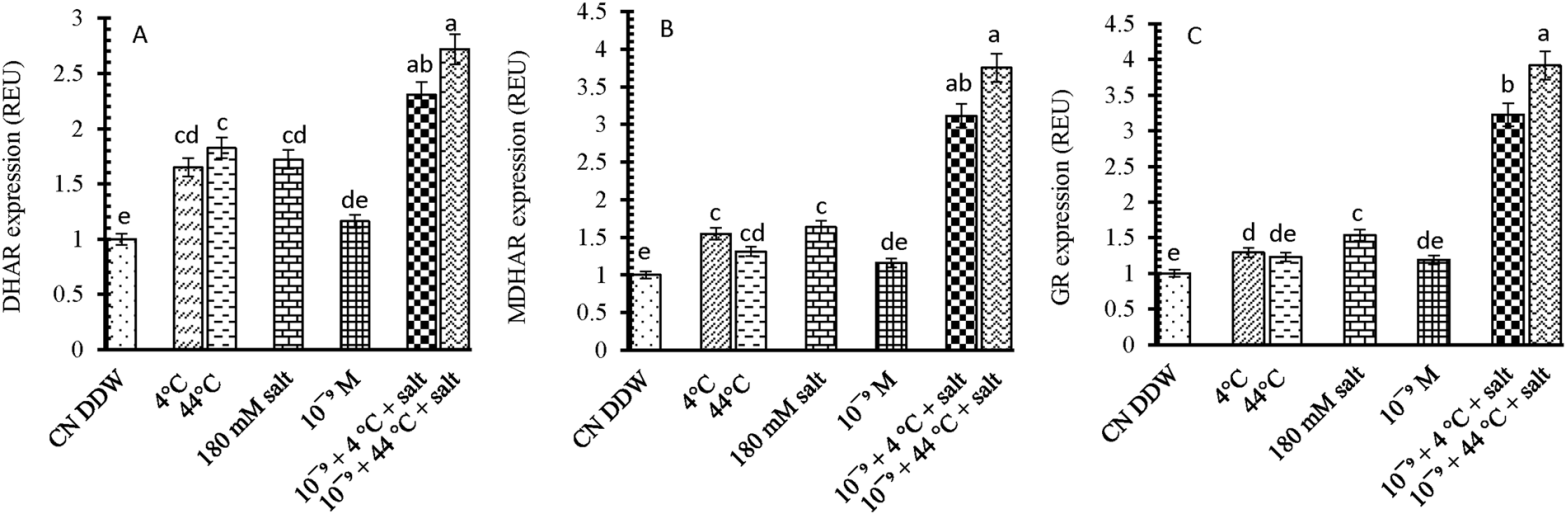
Effect of 28-HBL concentration (0, 10^−9^ M) on (**A**) DHAR, (**B**) MDHAR and (**C**) GR genes (REU relative expression unit) of *B. juncea* L. under combinations of extreme temperature (4 °C, 44 °C) and NaCl salt (180 mM) stress in laboratory conditions. Different superscripted alphabetical letters within a column indicate significant differences from each other in all combinations (Tukey’s test, p ≤ 0.05).

DHAR expression in seedlings of *B. juncea* L. was up-regulated by 64, 82 and 72% after exposure to individual 4 °C, 44 °C and salt treatments, respectively, compared with the control seedlings (Fig. 6A). The individual 10^−9^ M 28-HBL treatment resulted in some re-regulation, which increased to 172% when additionally exposed to 44 °C + salt.

MDHAR expression was up-regulated by 54, 31 and 63% exposure to individual 4 °C, 44 °C and salt treatments, respectively, compared with the control seedlings. Supplementation with 10^−9^ M 28-HBL combined with 44 °C + salt resulted in 275% up-regulation of MDHAR expression (Fig. 6B).

GR expression in seedlings of *B. juncea* L. was up-regulated by 29, 23 and 53% after exposure to individual 4 °C, 44 °C and salt treatments, respectively, compared with the control seedlings (Fig. 6C). The individual 10^−9^ M 28-HBL treatment increased GR expression by 18%, which was further up-regulated by 122% with 4 °C + salt and 191% with 44 °C + salt.

## Discussion

Extreme temperature and salt stress are major agricultural hazards that adversely affect crop production and may lead to imbalances in the antioxidant defense system and the amount of ROS, resulting in oxidative stress^4^. Plants have various defense mechanisms to react and acclimatize to extreme temperature and salt stress. The phytohormone brassinosteroid and its isoform 28-homobrassinolide can regulate the protective responses of plants; 28-homobrassinolide modifies antioxidant enzymes and non-enzymatic antioxidants under different stress conditions^44,45^. Exogenous application of 28-HBL is one method for improving temperature and salt tolerance in plants. Extreme temperature and salt stress reduced growth in *B. juncea* L. seedlings are supported by findings of Tajbakhsh, *et al*.^46^ in barley seeds and Smertenko, *et al*.^47^ in *Nicotiana tabacum* cells and in tall fescue^48^. Poor growth in plants exposed to extreme temperatures and salt is due to a reduced mitotic index, which affects overall plant growth^49^. In our study, the application of 28-HBL by pre-soaking seeds alleviated the detrimental effects of temperature and salt stress and increased plant growth, which supports the findings of Fariduddin, *et al*.^18^, Nassar^50^, Krasensky and Jonak^51^.

We attributed the poor growth performance of *B. juncea* seedlings under temperature and salt stress to the overproduction of ROS (O^2−^ and H_2_O_2_), which caused oxidative damage to lipids and increased MDA content, %EL and H_2_O_2_ content. H_2_O_2_ has a dual role in plants-at low concentrations, it acts as a signal molecule, while at high concentrations, it leads to oxidative damage^52^. In our study, H_2_O_2_ increased with low- and high-temperature stress and salt stress, which agrees with a similar study on *Triticum aestivum*^53^. Detoxification of H_2_O_2_ by 28-HBL has been studied in *B. juncea*^45^ and *Vigna radiata*^54^. A major impact of oxidative stress is a perturbed function or complete dysfunction of cellular membranes, which can increase cell permeability and ion leakage. In our study, electrolyte leakage was more pronounced under temperature and salt stress, which has been observed elsewhere: low temperature^55,56^, high temperature^57^ and salt stress^58^. Lipid peroxidation is one of the criteria for determining the impact of extreme temperature and salt stress; MDA is the end product of this reaction and is commonly considered a signal of oxidative stress^20,28,59^. Similar to our findings, Ogweno, *et al*.^26^ reported that different 28-HBL concentrations reduced MDA content and H_2_O_2_ content in tomato plants exposed to 40/30 °C temperature stress. Liu, *et al*.^60^ reported that the BR treatment in *Chorispora bungeana* protected cells from chilling stress by inhibiting MDA formation. Arora, *et al*.^61^ observed the mitigation of lipid peroxidation in *Zea mays* with exogenous supply of 28-HBL. Furthermore, Li, *et al*.^62^ reported that the BR treatment decreased MDA content in *Rubinia pseudoacacia* seedlings. In our study on *B. juncea*, the application of 28-HBL minimized the production of H_2_O_2_ and other ROS, which directly affected membrane lipids and %EL. How 28-HBL reduces H_2_O_2_, MDA production and electrolyte leakage is unclear. It is possible that 28-HBL enhances the scavenging capacity of antioxidants that might lead to low H_2_O_2_ production and ultimately reduce lipid peroxidation and electrolyte leakage, or that 28-HBL induces other endogenous phytohormones to directly or indirectly impart tolerance to plants through low ROS production^63^.

The potential of 28-HBL for mitigating the detrimental effects of temperature and salt stress on cellular organization was assessed in a cell viability/non-viability assay. Propidium iodide (PI) is a nucleus staining dye, which cannot pass through a viable cell membrane in root tips of *B. juncea* L. PI reaches the nucleus by passing through disordered areas of dead cell membranes and intercalates with the DNA double helix to stain cells red^64^. In our results, root cells of *B. juncea* L. exposed to temperature and/or salt stress stained red unlike the control cells treated with distilled water. Another method for checking cell viability uses fluorescein diacetate (FDA) which stains fluorescent green^65,66^. As cell membranes are permeable to esterified forms of FDA, it can enter cells freely and become entrapped intracellularly. After hydrolysis in intracellular spaces, the non-florescent FDA is converted to the green fluorescent metabolite in *Arabidopsis*^67^. In our study, reductions in green fluorescence in *B. juncea* L. seedlings treated with 28-HBL with or without temperature or salt stress suggest that 28-HBL has the potential to modulate oxidative stress during growth and development under different stress environments.

Soluble proteins can decline under extreme temperature and salt stress^68^. In our study, 28-HBL enhanced protein contents, which agrees with the findings of Bajguz^69^ where dose-dependent amelioration increased DNA, RNA and soluble protein contents in *Chlorella vulgaris*. Brassinolide action can be regulated via the receptor/ligand complex which binds to nuclear or cytoplasmic sites to regulate the expression of specific genes^70^. 28-HBL has induced some heat shock granules and increased thermotolerance under heat shock^71^. It has been reported that plant growth is dependent upon the synthesis of nucleic acids and proteins through transcription and translation^72^.

Stress tolerance induced by 28-HBL is also associated with increased activities of SOD, CAT, APOX, DHAR, MDHAR, GR and their expression^73^. Thus, the present study hypothesized that detoxification pathways in treated seedlings consist of several metabolic processes including the activation of antioxidant machinery. The enhanced SOD and CAT activities might efficiently scavenge harmful ROS, as indicated by significantly reduced MDA contents in *B. juncea* and observed by Li, *et al*.^62^, Mahesh, *et al*.^74^ and Huang, *et al*.^75^. Indeed, SOD is the first line of protection against ROS, which catalyzes the superoxide radical (O^2−^) to O_2_ and H_2_O_2_^76^. CAT is responsible for the conversion of H_2_O_2,_ a potent and detrimental oxidizing agent, to H_2_O and oxygen^77^. Further, Talaat^78^ reported that the ascorbate–glutathione pathway comprises a vital pathway for ROS dissipation in plant tissues and the alleviation of oxidative damage under abiotic stress is responsible for this detoxification pathway. Extreme temperature and salt conditions increased the activities of APOX and GR when supplemented with 28-HBL and increased the activities of APOX, MDHAR and DHAR. Increases in antioxidant enzyme activities in almost equal values confront the oxidative stress and play a vital role in preventing lipid peroxidation and eliminating ROS production. Further, this increase in enzyme activity reflects the efficient maintenance of the ascorbate and glutathione pool required for efficient detoxification via the ascorbate–glutathione cycle. Several reports have indicated that BR application enhances antioxidant enzyme activity under various stresses^17,79^. The augmentation of antioxidant activities by BRs seems to be the result of activation and *de novo* synthesis of enzymes regulated through the transcription and/or translation of specific genes^69^, which has added the potential for temperature and salt-stressed plants to resist oxidative stress. In addition, exogenous 28-HBL application improves antioxidant enzyme functioning and activation under temperature and salt stress^80^. Huang, *et al*.^75^ reported that 28-HBL could be conjugated with antioxidative enzymes to stabilize and modulate the structure and activity of enzymes.

Extreme temperatures and salt will impact antioxidant activities; much of the research is based on quantitative analysis with few reports on gene expression^81^. The literature on antioxidant gene expression in plants under combined temperature and salt stress supplemented with 28-HBL is limited. Extreme temperature and salt stress enhanced expression of SOD in this study while CAT expression increased in response to temperature stress in tomato^82^. Kaur, *et al*.^83^ reported that 28-HBL up-regulated gene expression of APOX, CAT and SOD in *B. juncea* under salt stress. Ara, *et al*.^84^ suggested that antioxidant enzyme activities and gene expression are associated with heat tolerance in the stems and roots of Cucurbita. Our results are similar to those of Jubany-Marí, *et al*.^85^, where maintenance and improvement of the ascorbate and dehydroascorbate cycles suggest the involvement of antioxidant defense system genes in the tolerance mechanism. Our results revealed that *B. juncea* L. seedlings treated with 28-HBL could combat oxidative stress, which might govern their protection from temperature and salt stress.

## Conclusion

The antioxidant system of *B. juncea* L. functioned at higher rates to prevent increased ROS formation under combined temperature and salt stress. This seemed evident in the evaluation of the extent of cellular damage, which was less remarkable when supplemented with 28-HBL. These results are in accordance with several studies conducted on different species, indicating that temperature and salt stress can induce the antioxidative system in *B. juncea* plants and that increased stress tolerance is correlated with decreasing oxidative injury.
